# Active Exercises in the Treatment of Locomotor Ataxia

**Published:** 1908-12

**Authors:** Theodore Toepel

**Affiliations:** Lecturer on Medical Gymnast.cs at the Atlanta College of Physicians and Surgeons, Atlanta, Ga.


### ACTIVE EXERCISES IN THE TREATMENT OF LOCOMOTOR ATAXIA.*

BY THEODORE TOEPEL, M. D., LECTURER ON MEDICAL GYMNAST.CS AT THE ATLANTA COLLEGE OF PHYSICIANS AND SURGEONS, ATLANTA, GA.

(Frenckel Method).

Before entering upon the treatment of Tabetic Ataxia it will be necessary for me to make brief mention of the various types of Tabetic Ataxia.

As regards locomotion the following groups may be distinguished:

1. The patient is able to walk about freely, without support, with or without the help of a cane, but his mode of walking is altered.

2. The patient has to lean upon the arm of a companion, with or without the support of a cane; walking alone is impossible.

*Read before Fulton County Medical Society, November 19, 1908.

3. Walking alone is no longer possible, but the patient can still stand.

4. Walking and standing are impossible. Moreover, at the beginning and at the end of this series should be mentioned on the one hand the so-called pre-ataxia stage and on the other the paralytic stage.

The numerous types of tabetic locomotor disturbance have several symptoms in common, which must be included in the definition of the term "ataxia."

The first is the integrity of the muscular power; the second is the close attention with which the patients follow with their eyes every single movement they make, whether in erect or recumbent position, the third and most important symptom is furnished by the fact that every ataxic movement becomes more pronounced if made with the eyes closed. There are no exceptions from this law, which applies to the erect as well as to the recumbent posture and can be used for the detection of ataxic symptoms in the majority of the patients who are still considered to be in the pre-ataxic stage.

It will be necessary to dwell upon the term "co-ordination" as it forms an important feature in the treatment of tabetic ataxia. The term "co-ordination" denotes the united action of a group of muscles in order to carry out an intended movement; the different degrees of contraction of the various members of a group of muscles may be styled "co-ordination of muscular contraction."

Whereas, it is possible by applying the electric current to stimulate a single muscle to contraction, the numerous individual and groups of muscles are at once combined for common action whenever an intended movement is to be made; in other words, the healthy organism knows only co-ordinate movements.

The factors which produce the co-ordination of the movements are: (1) the quality of the implicated muscle groups, (2) the magnitude of contraction which may be measured with dynamometer, (3) and the celerity of the movement in the joints, which latter depends on the swiftness of the contraction of the muscles.

*Co-ordinate movements can not be made unless the three aforesaid factors work well together.* The slightest irregularity on the part of one of them disturbs the whole movement.

Anatomy describes and names the individual muscles, while the physiology of movements deals with groups of muscles which are distinguished according to their various functions. The contraction of a single muscle (single in an anatomical sense) probably does never occur.

It appears that the will power influences not one muscle, but a group of muscles, which are combined for a common physiological purpose; at least it needs special training in order to enable the will power to influence an individual muscle.

It is by practice only that *dissolated innervation* is acquired, and with it proficiency in the finer kinds of manual labor, and in that kind of leg work which is required in some sports and acrobatic performances.

The muscles which belong to one physiological group differ both as to size and insertion; therefore every individual muscle of the group must, during the execution of an intended movement, enter into a certain, but of course, for each muscle, different state of contraction.

The treatment of locomotor ataxia is based upon the education of the central nervous system by means of repeated exercises, whereby it is enabled to receive sufficiently distant stimuli from the limbs as to their position and so on, although the available quantity of sensation is rather small.

It is necessary, of course, that the movements be attempted and carried out repeatedly and with great attention. Repetition enables the central nervous system to differentiate stimuli of minute intensity; its sensibility becames so great that often repeated slight stimuli act on it with the same force as rarer but much stronger impressions.

Theoretically, the transformation of an ataxic movement into a normal movement takes place in tabetic subjects according to the same laws as the acquisition in healthy persons of a complicated movement which acquires the differentiation of tactile impressions of minute strength. A certain minimum of sensation, however, is absolutely indispensable; complete anaesthesia precludes the application of the treatment by exercise, but fortunately, such cases are very rarely, if at all, met with.

The greater the loss of sensation the longer and the more difficult will be the treatment and the more uncertain the result.

The principal thing to do in this treatment is to find out for each limb the kind of exercise which is most apt to compensate the loss of sensation. If the loss of sensation has not been so great as to reduce the residue of sensibility below a certain minimum one usually succeeds in teaching the patient to stand or walk with his eyes closed; but the movements will always be more undecided than when they are made under the control of the eyes. We have no means of defining the minimum quantity of sensation which is necessary for producing a good result; but it is sufficient if the patient has a vague sensation of the active or passive movements which are performed by his limbs.

If the patient has a strong will and is able to give close and continued attention to the task he has to perform—a faculty which is much rarer than is commonly supposed—the result of the treatment will be excellent, with that unavoidable exception that the patient must keep his eyes on his movements as it were. Such patients are able, when lying on the back to carry out movements with perfect co-ordination and when walking they walk perfectly well with the single exception that there is more or less hypotonia and curving backward of the knees, or outward rotation of the thighs.

All healthy people move their limbs with a certain amount of steadiness and precision. Tabetics who have undergone the exercise treatment and have lost their ataxia show no obvious differences from the normal state, as long as their attention is not called away from the task they are just performing; but they show a great deal of hesitation and indecision when their attention is directed to something else. In such cases all depends on their determination and endurance; they must continue carefully and persistently to superintend their own movements for many months after their discharge, lest they will quickly return to their former ataxia; for this reason it is better for patients whose initiative and will power is weak to remain under observation for some months after the treatment proper has been concluded, until they have completely mastered their daily task and have acquired the habit of continuous and painstaking supervision of every detail of their movements. If the exercises are practiced daily with the same attention, the movements continue to gain precision.

The exercises may be classified according to the various functions of the affected limbs, and according to the manner and degree of ataxia.

As regards the various degrees and forms of ataxia, it makes it obligatory upon the physician to choose the exercises which are most suitable in each case, and intensity to the sequence, duration and intensity to the requirements of each individual case.

The exercises are divided into:

1. Those that are practiced in the recumbent position—in which the influence of gravitation and the necessity of keeping the equilibrium may be eliminated.

2. Those that take place when the patient is in a sitting position.

3. Those that are executed by the patient in an erect position.

4. Those which consist in various movements and evolutions carried on during walking.

Exercises in recumbent position.

Both legs are stretched out.

1. Flexion of one leg to different angles.

2. Same as 1, with abduction and adduction.

3. With sudden halting in the midst of the movement, the doctor giving the command.

4. The patient making a voluntary halt in the midst of flexion or extension.

5. Both legs—repeat 1, 2, 3 and 4.

Movements are made slowly and even. Repeat each exercise about 4 times at the beginning then increase the repetition slowly.

Keep leg moving in a vertical plane. In tabetic patient's legs can be flexed high on chest. Only flex as high so that heel touches other knee.

6. Repeat exercises 1-5, but keep heel off the couch.

7. Heel is placed on patella on other side then on different places of the tibia the ankle joint on the toes.

Strap garters with different colored pads to the legs.

8. Heels slide up and down the tibia, call halts at different places during the exercises.

9. The leg is flexed in the hip and knee to a right angle, then extend knee joint.

10. Both legs are flexed, both knees and inner malleoli remain in apposition.

11. With voluntary halts by the patient and by the doctor.

12. See saw, flexion and extension (alternate).

13. One leg is flexed while the other leg is abducted—extension of flexed and adduction of abducted.

14. Place heel on knee, follow course of tibia without touching down and back again.

15. The patient tries to place his heel in the hollow of the doctors hand, which constantly changes its position.

16. Exercises in bed with special apparatus. Bar—high and low. Board with notches which are numbered. Board with two rows of holes. Large and small rings—foot follows the circumference of this ring.

17. Repeat exercises, the eyes fixed on some object in the room. Assume sitting position and repeat exercises 1-17.

18. Sitting down and getting up. Erect position.

19. Walking slowly, watching closely the way he moves and correcting the outer rotation of his legs.

20. Steps of 6 inches, 14, 24 inches should be taken slowly—the patient dressed so that he can watch the movement well.

To get the distance have oil cloth with foot prints painted on. Use walking sticks. Fix eyes on prints and feet.

21. The long step, 28 inches in reserved for the last. This requires good control. The half step, 14 inches. The quarter step, 7 inches.

22. Single step forward (that is, after each step the feet are placed in apposition.)

23. Steps 1-2, 1-4, progression.

24. Combine steps of unequal lengths.

25. Stepping sideways.

26. Stepping backwards.

27. Change of direction—turning around.

28. Walking zig zag.

29. Walking with bent knees.

30 Walking on the narrow border.

30. Walking with objects in their hands—on the head.

31. The walking in cases of severe ataxia is with belt around thorax supported by two attendants—walking on the spot first exercise.

